# Effects of Hyperbaric (Non-Thermal) Sanitization and the Method of Extracting Pomegranate Juice on Its Antioxidant and Antihypertensive Properties

**DOI:** 10.3390/antiox13081009

**Published:** 2024-08-19

**Authors:** Gieraldin Campos-Lozada, Jonathan Hernández-Miranda, Leonardo del Valle-Mondragón, Araceli Ortiz-Polo, Gabriel Betanzos-Cabrera, Gabriel Aguirre-Álvarez

**Affiliations:** 1 Institute of Agricultural Sciences, University Autonomous of Hidalgo State, Av. Universidad No. 133, Col. San Miguel Huatengo, Santiago Tulantepec C.P. 43775, Hidalgo, Mexico; ca409778@uaeh.edu.mx; 2 Elviruch S.A. de C.V. Parque Industrial, Avenida Coatepec 520 ISB, Bodega 35C, San Bartolomé Coatepec, Huixquilucan de Degollado C.P. 52770, Mexico; jonathanhernandezm93@gmail.com; 3 Departamento de Farmacología, Instituto Nacional de Cardiología Ignacio Chávez, Juan Badiano No. 1 Col. Sección 16, Tlalpan, Ciudad de México C.P. 14080, Mexico; leonardo.delvalle@cardiologia.org.mx; 4 Instituto de Ciencias de la Salud, Área de Nutrición, Universidad Autónoma del Estado de Hidalgo, Ex Hacienda la Concepción s/n. Carr. Pachuca-Tilcuautla, Tilcuautla C.P. 42060, Hidalgo, Mexico; araceli_ortiz4208@uaeh.edu.mx (A.O.-P.); gbetanzo@uaeh.edu.mx (G.B.-C.); 5 Uni-Collagen S.A. de C.V., Arnulfo González No. 203, El Paraíso, Tulancingo C.P. 43684, Hidalgo, Mexico

**Keywords:** pomegranate juice, hyperbaric sanitization, antioxidants, phenols, flavonoids, anthocyanins, ACE

## Abstract

Pomegranate (*Punica granatum* L.) is considered a functional food due to its polyphenol content that benefits the body. The type of processing the fruit undergoes is important, as this also influences the concentrations of these compounds. The pomegranate juice was extracted by two methods: manual extraction using a manual juicer through heat treatment in a water bath (Man-P), and extraction through mechanical pressing using Good Nature X-1 equipment and hyperbaric sanitization (Mech-Hyp). Bromatological analyses showed significant differences (*p* ≤ 0.05) between the two treatments. When subjected to hyperbaric sanitization, the juice showed higher concentrations of moisture, soluble solids, protein, and carbohydrates. In an antioxidant analysis, the ABTS radical showed no significant difference in the treatments, with 96.99% inhibition. For the DPPH radical, the sample with the highest inhibition was Man-P with 98.48%. The determination of phenols showed that there was a higher concentration in juice that underwent pasteurization (104.566 mg GAE/mL). However, the Mech-Hyp treatment exhibited a minor concentration of phenols with 85.70 mg GAE/mL. FTIR spectra revealed that the functional groups were mainly associated with carbohydrates. Regarding ACE inhibition, it was observed that the Man-P and Mech-Hyp juices showed greater inhibition of enzyme in hypertensive patients compared to normotensive patients. This activity can be attributed to the mechanisms of action of antioxidant compounds. Both extraction methods manual and mechanical pressing resulted in increased antioxidant and antihypertensive activity. The antioxidant compounds accompanied by adequate sanitation were decisive in an antimicrobial analysis, since no pathogenic microorganisms were observed in the juices.

## 1. Introduction

The pomegranate (*Punica granatum* L.) belongs to the Lythraceae family and is a small tree with deciduous leaves. Its fruit is considered a berry with a hard shell (pericarp) containing seeds, each of them surrounded by fleshy and juicy pulp (arils) which in turn is attached to a soft and spongy tissue (mesocarp). Once cut, the fruit does not continue to ripen, so it is a non-climacteric fruit [1]. Mexico produces 6800 tons of pomegranate per year [2]. It is mainly produced in Hidalgo, Morelos, and Oaxaca, and the season in which it is produced is the months from July to October. It is considered a functional food because of its content of polyphenols that benefit the body [3]. The antioxidant activity of these compounds is due to the scavenging or neutralization of reactive oxygen species (ROS), as well as the inhibition or activation of signaling pathways and modulation of gene expression [4]. Pomegranate arils, which are the edible part of the fruit, can be consumed fresh or used to prepare gelatinous beverages, jams, and juices, among other things, and they constitute approximately 50% of the total fruit. Arils have a color that varies among crops from white and pink to red and dark red [5]. Pomegranate juice also contains fructose, sucrose, and glucose as well as small amounts of amino acids such as proline, methionine, and valine. It also possesses simple organic acids such as ascorbic acid, citric acid, and fumaric acid. Pomegranate skin and juice are rich in polyphenols, the most prominent of which are tannins and flavonoids. These components are noted for their potent antioxidant and preservative functions [6].

The way fruit is processed is important because it influences the concentrations of compounds [7]. Currently, the market has introduced cold-pressed juices; this emerging technology preserves the bioactive compounds in fruits, avoiding their degradation [8]. Heat treatment reduces nutritional compounds and modifies physicochemical properties. This is why heat treatment is being replaced by non-thermal sanitization treatments such as high-pressure processing (HPP) to improve the shelf life of juice without affecting its organoleptic properties [9]. The pasteurization process in the production of juices and beverages involves the application of heat treatments to eliminate pathogens. However, this step significantly affects some bioactive compounds and consequently the antioxidant activity of the juice. The growing demand for high-quality products that maintain their compounds and properties has promoted interest in emerging technologies such as hyperbaric sanitation. This is a form of non-thermal sanitization processing where high pressures ranging from 100 to 1000 MPa are applied. This process allows microbial inactivation and protects the physicochemical, bioactive, and sensory properties of juices, in addition to extending their shelf life [10]. Currently, there is a high rate of consumption of fast food, canned foods with preservatives, and beverages with high sugar content and small amounts of antioxidants [11]. For this reason, cold-pressed juices have been introduced in the market to provide a larger amount of compounds without pasteurization [8]. Phenolic compounds are the main antioxidants that should be present in the human diet. These compounds have become of interest to researchers. Several studies support that these phenolic compounds have vasodilator, vasoprotective, and cardioprotective effects, among others [12]. Worldwide, about one billion people suffer from hypertension. At the molecular level, several factors are involved in this pathophysiology. One of them is oxidative stress, which causes excessive production of vascular reactive oxygen species (ROS) that play an important role in the control of endothelial and vascular function [13]. On the other hand, they also attenuate platelet aggregation and decrease hypertension, improving vascular function [14] and providing atheroprotective inflammatory effects on the vascular wall [15]. It is considered that the health problems mentioned above are related to reductions in the consumption of bioactive compounds that can be degraded when food products are subjected to thermal treatment. The importance of this study is the use of emerging technologies, such as hyperbaric sanitation, to evaluate the antioxidant properties of the bioactive compounds of pomegranate juice and their effect on serum from hypertensive people. 

## 2. Materials and Methods

Pomegranate fruit of the Wonderful variety was purchased in Tasquillo, Hidalgo, Mexico.

### 2.1. Extraction Process 

#### 2.1.1. Extraction of Aril Juice Using a Manual Method 

The fruits were washed with soap at 1% and water. The arils were manually separated from the peel and then chemically sanitized for 15 min. Subsequently, the juice was extracted using a manual juicer (WIKHOSTAR, No. k051, USA) The juices were bottled in hermetically sealed 250 mL opaque white polypropylene bottles. The juice was pasteurized in a water bath (Thermo Scientific No. TSU-GP20 Series) at 60 °C for 30 min and kept refrigerated at 5 °C. Samples were described as having undergone “manual extraction followed by pasteurization” (Man-P). 

#### 2.1.2. Cold Pressing Extraction of Juice from Arils 

The fruits were washed, and the arils were manually separated. Arils were subjected to a chemical sanitization treatment (Suma frescor chlorine, 80 ppm). The juice was extracted using Good Nature X-1 equipment, which was previously washed (0.5% *v*/*v* Diverdet detergent) and sanitized with sodium hypochlorite (NaClO) solution at 80 ppm. The arils were placed in a press bag (Press Bag—Wide Weave) and pressed with a force of 1800 PSI for 40 s. Three high-pressure cycles were used to complete the juice extraction. The samples were bottled in hermetically sealed 250 mL opaque white polypropylene bottles. The packaged samples were subjected to high-pressure sanitization treatment (Hyperbaric 55, España) in which one cycle of operation was performed at 5000 bar for 5 min at 12 °C. The samples were kept at 5 °C and labeled as “mechanical extraction with hyperbaric sanitization” (Mech-Hyp).

### 2.2. Physicochemical Characterization

#### 2.2.1. Bromatological Analysis

The following analyses were carried out according to the AOAC (2000): moisture (950.27), fat (986.25), protein (999.12), ash (923.03), fiber (992.21), and total soluble solids (932.12).

#### 2.2.2. pH 

The pH measurements were performed in triplicate following method 981.2 described by the AOAC (2000) using a calibrated potentiometer (HANNA Instruments, model HI 2210; Limena, Italy). 

#### 2.2.3. Color Determination 

The color of the sample juices was determined using a Konica Minolta colorimeter (model CR-400/410, Foster City, CA, USA). A total of 5 mL of each sample was placed in a white container and analyzed by using a CieLab scale.

#### 2.2.4. Fourier Transform Infrared Spectroscopy (FT-IR) 

The absorption spectra of the juices were obtained using Fourier trans-form infrared spectroscopy (FTIR) (Perkin Elmer; Boston, MA, USA). Samples were placed in intimate contact with the diamond crystal by applying a loading pressure and scanned over a range from 400 cm^−1^ to 4000 cm^−1^. The results were analyzed using the Spectrum^TM^ 10 software (Perkin Elmer, Boston, MA, USA). 

#### 2.2.5. Microbiological Analysis

The determination of *Salmonella* and *E. coli* was carried out according to Mexican legislation (NOM-210-SSA-2014). The NOM-11-SSA-1994 and NOM-092-SSA-1994 methods were followed for the determination of molds and yeasts in food, and the aerobic bacteria count (aerobic mesophiles), respectively. The samples were incubated in 90 × 15 mm Petri dishes for 24 h in an oven (FELISA model FE-131; Zapopan, Jalisco, Mexico) at 37 ± 2 °C. The results were expressed as the presence or absence of microorganisms.

#### 2.2.6. Total Phenols Determination 

The determination was carried out using the Folin–Ciocalteu method; 0.5 mL of sample was mixed with 2.5 mL of diluted Folin–Ciocalteu reagent (1:10) (Sigma Aldrich Mexico) and maintained for 5 min in complete darkness. Then, 2 mL of 7.5% sodium carbonate solution was added and homogenized. After 2 h of reaction, the absorbance was measured in a spectrophotometer (JEAWAY, Model 6705) at a wavelength of 760 nm. The results were expressed in mg of gallic acid equivalents (mg GAE). A calibration curve was previously prepared from a stock solution of gallic acid at a concentration of 1000 mg/L.

#### 2.2.7. Flavonoids Determination

First, 2 mL of aluminum trichloride methanolic solution at 2% (AlCl_3_) (Fermont, chemical products Monterrey, Mexico) was mixed with 2 mL of the sample and kept in the darkness for 20 min. The absorbance was read at 415 nm in a spectrophotometer (JEN-WAY, model 6705). The results were expressed in milligrams of quercetin equivalent (mg/EQ). A calibration curve was prepared from a quercetin stock solution at 1000 mg/L concentration.

#### 2.2.8. Determination of Total Monomeric Anthocyanin Content Using the pH Differential Method for Total Anthocyanins (AT)

This method was performed by using the pH difference. Two solutions were used: (1) potassium chloride (pH 1.0) and (2) sodium acetate (pH 4.5). In a test tube, 2 mL of solution 1 and 500 μL of juice were mixed (15 min, 25 °C). In another test tube, 2 mL of solution 2 and 500 μL of juice were mixed (15 min, 25 °C). The same step was performed for the sodium acetate buffer. Each tube was read at two absorbances 520 nm (tube with buffer 1) and 700 mn (tube with solution 2) in a spectrophotometer (Jenway Genova, Model 6705, Bibby Scientific; Stafford, UK) [16]. The final absorbance was calculated with the following equation:

$$A={(A}_{520}-A_{700\;nm}{)}_{pH\;1.0}-{(A}_{520}-A_{700\;nm}{)}_{pH\;4.5}$$

The concentration of monomeric pigments in the extract was expressed as cyanidin-3-glucoside.

$$Anthocyanin\;pigment\;(cyanidin-3-glucoside\;equivalents,mg/L)=\frac{A\times Mw\times DF\times 1000}{\left(\varepsilon \times 1\right)}$$

Here:

Mw (molecular weight) = 449.2 g/mol for cyanidin-3-glucoside (cyd-3-glu).
DF = dilution factor established in D.
1 = pathlength in cm.
ε = 26,900 molar extinction coefficient, in L mol^−1^ cm^−1^, for cyd-3-glu.
The factor for conversion from g into mg was 1000.
A = absorbance.
Mw = molecular weight.
DF = dilution factor.

#### 2.2.9. Determination of ABTS Radical Scavenging

An ABTS (2, 2′-Azino-bis [3-ethylbenzothiazoline-6-sulfonic acid]) radical solution was prepared according to the method of Re et al. [17]. Firstly, 2.45 mM potassium persulfate was mixed in a 1:1 *v*/*v* ratio with 7 mM of ABTS (Sigma-Aldrich; St. Louis, MI, USA). The mixture was stirred at room temperature for 16 h in the darkness. The ABTS solution was diluted to 0.70 ± 0.02 at 734 nm using ethanol. Then, 2 mL of the sample and 1 mL of stabilized radical ABTS solution were mixed, and after 6 min, the sample was read at 734 nm in a spectrophotometer (Jenway Genova, Model 6705, Bibby Scientific; Stafford, UK). The following formula was used to determine the percentage inhibition:

$$Inhibition\;\%=\frac{initial\;absorbance-final\;absorbance}{initial\;absorbance}\times 100$$

#### 2.2.10. Determination of DPPH Radical Scavenging

For decolorization of the DPPH (2,2-diphenyl-1-picrylhydrazyl) radical, 0.5 mL of the sample was mixed with 2.5 mL of 1.9 × 10-4 mol/L methanol DPPH solution (Sigma-Aldrich; St. Louis, MI, USA). The mixture was kept in the dark for 30 min and then read in a spectrophotometer (Jenway Genova, Model 6705, Bibby Scientific; Stafford, UK) at 515 nm. The following formula was used to determine the percentage inhibition:

$$Inhibition\;\%=\frac{initial\;absorbance-final\;absorbance}{initial\;absorbance}\times 100$$

### 2.3. Inhibition of Angiotensin Converting Enzyme (ACE) Activity by Pomegranate Juice

For the analysis of angiotensin-converting enzyme (ACE) activity, as well as the inhibition of its activity in the processed juices, a commercial assay kit was used (Angiotensin 1 Converting Enzyme “ACE” Activity Assay Kit—Fluorometric /Sigma Aldrich, Cat. CS0002-1KT, Batch. 0000128503/St. Louis, MO, USA). The assay was based on the cleavage of a synthetic fluorogenic peptide. The measured fluorescence was directly proportional to the ACE activity present. A fluorescence spectrophotometer (Cary Eclipse, Varian Inc., Mulgrave, VIC, Australia) with an excitation wavelength of 320 nm and emission wavelength of 405 nm was used for the experimental work. The serum was donated by the National Institute of Cardiology in Mexico City and corresponded to serum from female patients between 56 and 65 years of age, with mean blood pressure levels of 152/92 +/− 13.25 mmHg. The serum was analyzed to identify whether the sample belonged to normotensive or hypertensive patients. From this evaluation, there were selected 40 samples in total (20 samples from normotensive patients and 20 samples from hypertensive people). The normotensive group was distributed into two groups (Man-P and Mech-Hyp) with 10 replicates for each group. The same distribution was carried out for hypertensive samples. Enalapril at a concentration of 50 µM was used as a control. The results were expressed in nmol of ACE inhibited per minute (nmol/min). For each juice sample (0.95 µL/mL), two reaction tubes were prepared in duplicate: one with 10 serum samples from hypertensive patients, and the other with 10 serum samples from normotensive patients. Serum from normotensive patients and from another group of hypertensive patients was included as a control. Lisinopril at 50 µM was included as a specific ACE inhibitor. A standard curve was prepared in a range of 0.0 to 0.8 nmol in a volume of 100 µL, and in duplicate the corresponding amount of standard and assay buffer were mixed in duplicate. A kinetic curve was constructed by plotting the measured fluorescence for each sample in relative fluorescence units (RFUs) versus time (in minutes), and the linear regression equation was determined for each sample. Enzyme activity was calculated using the linear regression slope of the standard curve and the linear regression slope of the sample kinetic curve to transform the sample values from RFU/min to nmol/min (units) according to the following equation:

Sample Enzyme Activity(nmol)min=[SampleSlope]StdSlope×DF

where:

StdSlope = slope of the standard curve subtracted from the blank (RFU/nmol);
DF = dilution factor (if the sample was not diluted, the DF value was 1);
SlopeSample = slope of the sample curve subtracted from the blank (RFU/min).

### 2.4. Statistical Analysis 

The experiments were conducted using a completely randomized design with three replicates. In the case of the Angiotensin Converting Enzyme Inhibition test, 10 replicates per treatment were considered. Data were analyzed using one-way analysis of variance (ANOVA), and different means were separated using the Tukey test (α = 0.05) with SPSS software (SPSS Inc., Chicago, IL, USA) v25. 

## 3. Results and Discussions

### 3.1. Bromatological Analysis

Pomegranate fruit contains vitamins (A, E, C, B6, B5, and K) and minerals such as copper, potassium, iron, and sodium. It also contains trace elements (magnesium, phosphorus, zinc, and selenium), fat, proteins, and carbohydrates [18]. Table 1 shows the bromatological analysis of the juices expressed on a wet basis. Mech-Hyp had the highest moisture concentration, around 92%, while the Man-P treatment showed a value of 88%. Priyanka et al. [19] conducted a comparative analysis of two types of pomegranates, Ganesh and Arakta, and found their moisture contents were 80.50% and 79.80%, respectively. For total soluble solids, the Mech-Hyp treatment showed a higher concentration (13%), and the Man-P sample had a mean value of 11%. Vardin and Fenercioǧlu [20] conducted a study on the clarification of pomegranate juice using the whole fruit. They found that the percentage of total soluble solids after pressing was 14.6%. In a study by Conidi et al. [21], juices were obtained using only the soft, fleshy structure that covers the seeds, which is called the sarcotesta. It contained approximately 15% solids, unlike juices obtained from the whole fruit, which had approximately 16% to 17%. Gil et al. [22] analyzed processed pomegranate juices from pomegranate arils using manual pressing. They reported 15.5% soluble solids. Meanwhile, juices obtained from frozen arils were previously stored for 9 months at −20 °C and contained 16.6% soluble solids. The Ganesh variety had 15% total soluble solids, while the Arakta variety had 14%; the fat content of Ganesh and Arakta was 0.2% and 0.25%, respectively [19]. 

The percentage of fat did not show significant differences (*p* > 0.05) in the range from 0.22% to 0.29% among the samples evaluated. This amount was due to the fact that the seeds were not fractured, as this is where fat is deposited. The ash concentration was higher in Mech-Hyp at 0.26%, while Man-P showed 0.20%. Mech-Hyp had resulted with the highest protein percentage of 0.31% while Man-P presented 0.17%. The carbohydrate content is important from a nutritional point of view because of its energetic contribution to the body. The treatments showed significant differences (*p* ≤ 0.05) with respect to the carbohydrate percentage: Mech-Hyp had a higher content of around 12%, while Man-P had 10%. In the literature, the carbohydrate percentage was 14% for Ganesh and 14.5% for Arakta. In addition, the mineral content was 0.8% in the Ganesh variety and 0.9% in Arakta. Regarding the amount of protein, the Ganesh and Arakta cultivars were reported to have 1.6% and 1.5%, respectively. The results obtained in the bromatological analyses in this work were similar to those reported by Priyanka and coworkers [19]. Regarding the fiber content, the results showed that there was no presence of fiber in the juices obtained. These bromatological results confirmed the suitability of the pomegranate juices for human consumption.

### 3.2. pH Value

Pomegranate juice contains organic acids, such as ascorbic, citric, and malic acids, among others. Due to the presence of these acids, the pH of the samples was recorded as 2.70, so there were no significant differences (*p* > 0.05) between the treatments. Priyanka et al. [19] conducted a comparative evaluation of the physicochemical properties of pomegranate fruits of the Ganesh and Arakta pomegranate varieties. They obtained pH values of 3.2 and 3.1, respectively. Mousavi and coworkers [23] reported that the pH of unfermented juice with lactic acid bacteria was around 3.09. After fermentation, there was an increase in the pH to 5.6. This means that the fermentation process also has an impact on the acidity of the juice. Vardin and other authors [20] carried out a study on the development of pomegranate juice processing using whole and clarified fruit with gelatin, polyvinylpolypyrrolidone (PVPP), and natural sedimentation. In their study, they analyzed the pH and identified that it was in the range of 2.88 to 3.03. 

### 3.3. Color Determination 

The CIE La*b* color scale has been widely used to describe colors that correspond to subjective perception and the interpretation is expressed using the following coordinates L = black/white (+ indicates white, − indicates black), a* = +red, −green and b* = +yellow, −blue [24]. According to the results shown in Table 2, the color evaluation gave a luminosity value of 16.28 and 16.91 for Man-P and Mech-Hyp, respectively. Therefore, it was observed that there were no significant differences since the representative color of the pomegranate fruit was red, and its luminosity was low. The values of coordinate a* showed the degree of coloration of the treatments, Man-P presented a value of 15.19 while Mech-Hyp presented a value of 13.43. The b* coordinate showed significant differences in each of the treatments, with Mech-Hyp presenting the highest coloration with 2.02, followed by Man-P with 3.34. It was considered that the significant differences (*p* ≤ 0.05) presented by the treatments were due to the juice extraction methods, since applying greater pressure on the fruit allowed the extraction of a greater amount of compounds such as anthocyanins [25]. Cervantes and collaborators [26] reported CieLab values of L = 30.81, a* = 53.78, and b* = 9.08 in pomegranate juice obtained with a manual juicer and pasteurized with electrical pulses. Arendse et al. [27] studied the Wonderful variety and reported the following results: the extraction methods included extracting juice with a juice extractor without crushing the seeds, yielding a brightness of 19.27, a* = 18.00, and b* = 2.57. For juice that was extracted by using a blender and crushing the seeds, they reported L= 24.02, a* = 30.63, and b* = 10.09. In juice extracted from half of the fruit using a manual juicer, they obtained L = 25.81, a* = 32.67, and b* = 11.80. The results obtained in this research agree very well with those obtained by Arendse and collaborators because the juices were similar to those extracted using a juice extractor without crushing the seeds. The color of food is due to natural substances that have biological functions in tissues. These are known as pigments or colorants and are molecules with organic functional groups [28]. 

### 3.4. Fourier Transform Infrared Spectroscopy (FT-IR) 

**Figure 1:**
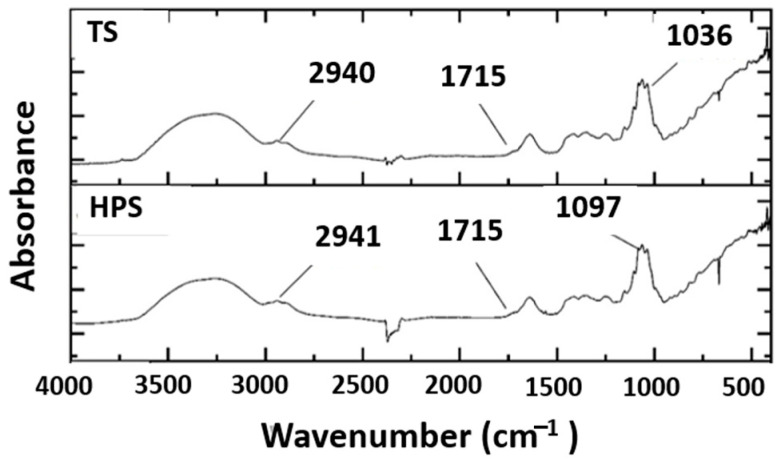
This figure shows the spectra of the functional groups identified in the juices.

Figure 1 shows the FTIR spectra of pomegranate juice obtained using the traditional method and cold-pressed pomegranate juice. The following bands were identified from left to right in both samples: 2940 cm^−1^, 1715 cm^−1^, 1097 cm^−1^, and 1036 cm^−1^. As described by Corripio et al. [29], bands at 1200–900 cm^−1^ are associated with carbohydrates in pomegranate juice. On the other hand, Leopold et al. [30] mentioned that the fingerprint region of 900–1400 cm^−1^ corresponds to glucose, fructose, and sucrose. When these three carbohydrates are present in the same solution, a strong overlapping of bands is generated and this makes it difficult to identify them individually. The bands in the 900–1153 cm^−1^ region were assigned to the C-O and C-C stretching modes while those in the 1400–1199 cm^−1^ region were due to the O-C-H, C-C-H, and C-O-H bending vibrational modes of the carbohydrates. In both samples, the same characteristic functional groups of the juices were detected. This means that the effect of the extraction methods and sanitation methods did not modify the structure of the samples, which maintained their fingerprints. 

### 3.5. Microbiological Analysis

The Man-P sample was pasteurized at 60 °C for 30 min, while the Mech-Hyp sample was subjected to hyperbaric sanitation. Both techniques are intended to eliminate pathogens because they affect the quality of the food. The sanitization methods used in this work were successful and there was no contamination by microorganisms from day 0 to day 60. The results were expressed as the presence or absence of microorganisms and indicated the adequacy of the pasteurization process. The presence of antioxidant compounds in the juice was considered to have an inhibitory effect on microorganisms. Bertucco and Vetter [31] mentioned that high-pressure technology has been used in different industries, highlighting its use in food production in order to achieve sterilization in fruit juices. The growing demand for fresh and minimally processed products has led to the use of non-thermal sanitization technologies, such as high-pressure processing (HPP), for fruit and vegetable processing to ensure the microbial safety and nutritional quality of foods. This hyperbaric technology includes hydrostatic pressures of 100 to 900 MPa [32], reducing enzymatic activity, maintaining its organoleptic properties, and preventing the growth of microorganisms [33]. The elimination or reduction in the presence of pathogenic microorganisms is supported by the principles of operation of high pressures. Firstly, the temperature increases by 2 to 3 °C when the applied pressure increases by 100 MPa. Secondly, any reaction, phase transition, or conformational change is favored by pressure, which is accompanied by a decrease in volume. Finally, the products are compressed due to pressure regardless of the size and geometry of the product because the transmission of pressure to the core does not depend on mass or time [34].

### 3.6. Phenols, Flavonoids, and Anthocyanins Content in Juice from Arils 

The antioxidant activity of pomegranate fruit is attributed to polyphenols this activity is related to their redox properties as oxygen donors, metal chelators, reducing agents, and oxygen scavengers [35]. The polyphenol content of pomegranate juice is approximately 0.2 to 1.0%, and includes hydrolysable tannins, ellagic acid derivatives, and flavonoids, depending on the variety of the fruit [36]. The most important polyphenol is punicalagin, which belongs to the ellagitannin family and is considered to be responsible for more than half of the antioxidant effect of pomegranate juice [21]. 

#### 3.6.1. Phenols

**Figure 2:**
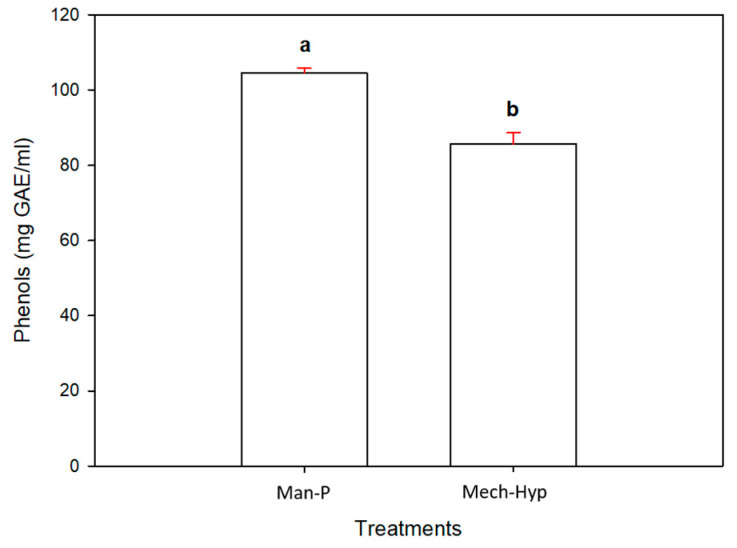
Phenol content in the juices. Different letters represent significant differences (*p* ≤ 0.05). Error bars represent ±1 SD of three replicates.

Figure 2 shows that the Man-P treatment had a higher number of phenols with 104,566 mg GAE/mL, followed by the Mech-Hyp treatment with 85.67 mg GAE/mL. It is important to mention that the significant differences (*p* ≤ 0.05) between samples may have been due to the concentration of water in them. According to the moisture content described in Table 1, the Mech-Hyp sample had a larger amount of water. This could dilute the number of phenols. Mechanical pressing caused a greater extraction of water than manual pressing (Man-P). Previous research [37] analyzed the antioxidant potential of extracts from pomegranate arils of Sri Lankan cultivars obtained through manual pressing. They found that there were variations in phenolic compounds among the samples. Akhavan and coworkers [38] studied the phenolic compounds and antioxidant activities in ten juices from arils grown in Iran. Their extraction method involved using a homemade press, and they found a total phenol content between 0.22 and 1.267 mg GAE/mL. The Daya cultivar had a higher phenolic content of around 2.390 mg GAE/mL, and the Indian cultivar had a content of 1.199 mg GAE/mL. In addition, these authors mentioned that phenolic compounds are one of the main groups acting as primary antioxidants, and especially as free radical scavengers. Esposto and coworkers identified significant differences between concentrated juices in the range of 1.379–3.748 mg GAE/mL and non-concentrated juices in the range of 1.632–2.736 mg GAE/mL [39]. Tezcan [40] evaluated the phenolic compounds in several commercial pomegranate juices. These compounds ranged from 2.602 to 10.086 mg/mL. Gözlekçi and collaborators [41] analyzed pomegranate juices obtained from pomegranate arils of different Turkish pomegranate cultivars (“Lefan”, “Katirbasi”, “Cekirdeksiz-IV”, and “Asinar”) using a manual press. They found that the phenolic content varied between 12,295 mg/mL and 15,515 mg/mL. Derakhshan et al. [42] analyzed three varieties of pomegranates in the markets of the Iranian provinces of Natanz, Shahreza, and Doorak using seeds, peels, and juices that were dried in an incubator at 30–40 °C. Dried samples were powdered with a grinder following an extraction with 80% ethanol and stored at room temperature. The Doorak variety showed 12.4 mg GAE/mL in juice, the Shahreza variety showed 15.8 mg GAE/mL in juice, and the Natanz variety showed a concentration of 23.8 mg GAE/mL in juice. These results appeared to be lower than those reported in this research. This could be attributed to the variety of the fruit, the place of harvest, and the drying treatment used. Mena and collaborators [43] analyzed pomegranate juices of the “Maravillosa” and “Mollar de Elche” varieties collected in the Alicante region. They obtained the juices of each cultivar through pressure with a laboratory pilot press and reported a total phenol content of 288 mg GAE/100 mL for Mollar de Elche and 390 mg GAE/100 mL for Wonderful. However, Rios-Corripio et al. [44] reported higher phenol contents than those in this research. They analyzed the antioxidant characteristics of pomegranate drinks fermented with *Saccharomyces cerevisiae* yeast and unfermented aril juice using Turmix equipment. They found that the content of phenolic compounds was approximately 393.78 mg GAE/100 mL. Comparing the results reported in this research with those reported in the literature, it was observed that the difference between the ranges of concentration of phenolic compounds was mostly due to the variety of the fruit and the extraction method. However, it is also known that the concentration of phenols depends on the type of heat treatment used, the oxidation state, and possible interactions with other compounds in the juice [45]. Alper et al. reported that pomegranate juice subjected to a temperature of 50 °C showed a decrease in the presence of these compounds [46]. This thermal pasteurization damaged the nutritional and physicochemical properties. The use of temperatures below 70 °C contributes to keeping the bioactive compounds in good condition and ensures microbial safety [47]. Subjecting pomegranate juice to temperatures higher than 90 °C reduces the content of total phenolic compounds compared with unpasteurized juice under refrigeration treatment [48]. In comparison with pasteurization, other emergent technologies such as UV radiation do not affect the compounds [49]. Regarding the use of high pressure, several authors mentioned that the use of this technology keeps the phenolic compounds, anthocyanins, and antioxidant capacity in the juices stable [50,51]. This is why the use of technologies such as hyperbaric sanitization uses low temperatures, which allows for the preservation of compounds and the elimination of pathogenic agents.

#### 3.6.2. Flavonoids

**Figure 3:**
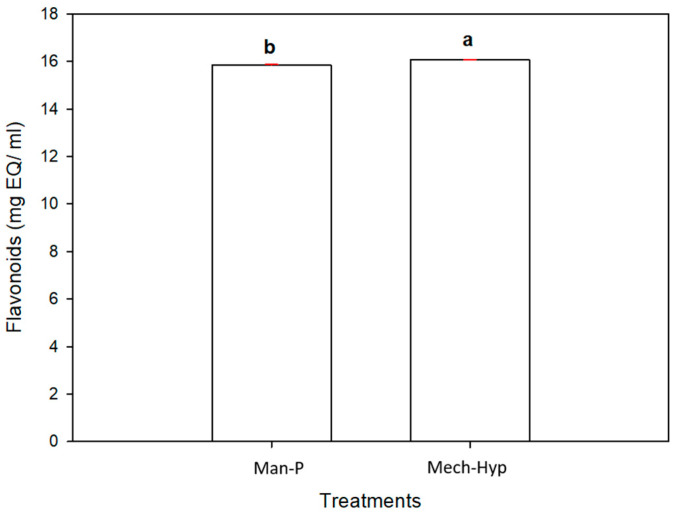
Flavonoid content in the pomegranate juices. Different letters represent significant differences (*p* ≤ 0.05). Error bars represent ± 1 SD of three replicates.

Flavonoids are natural substances formed by two phenyl rings and a heterocyclic ring [52]. Previous studies [53] mentioned that more than 8000 different flavonoid compounds have been identified. Their main functions in plants are to protect them against ultraviolet radiation, pathogens, and predators and to avoid oxidative stress, among others. Figure 3 shows the results obtained for the Mech-Hyp and Man-P treatments. There were significant differences (*p* ≤ 0.05) in the concentrations of flavonoids, which were 16.06 (mg EQ/mL) and 15,850 (mg EQ/mL), respectively. Li and collaborators [54] analyzed the pomegranate juices of 10 Chinese cultivars using only the arils for extraction with a home juicer. They found a total flavonoid concentration ranging from 0.045 to 0.335 mg EQ/mL. Fahmy and coworkers [55] reported a flavonoid concentration from 0.234 to 0.504 mg catechin equivalent/mL in pomegranate juice. El Kar and coworkers [56], analyzed nine pomegranate arils juices obtained with a blender from nine Tunisian ecotypes. The flavonoid contents were reported to be between 0.135 and 0.636 mg EQ/mL for the Gabsi and Tounsi varieties, respectively. These results were lower than the amounts reported in this work. According to Zhao and collaborators [57], the concentration of these compounds in fruits depends on the species, type of crop, part of the plant, climatic conditions, and degree of maturity. Aloqbi et al. [58] reported the flavonoid content in the juice of Spanish pomegranate arils. The juice was freeze-dried and stored at −80 °C, and the phenol content reported was 31.5 mg catechin/60 mL based on the dry weight of the pomegranate arils. Regarding the sanitization process, Farahmand et al. [59] evaluated the effects of pasteurization in pomegranate juice. They did not observe a significant effect on the total phenolic content, but there was a reduction in the total content of total flavonoids and anthocyanins. This trend and the behavior of the results agree very well with those obtained in this study using mechanical extraction and hyperbaric sanitization (Mech-Hyp).

#### 3.6.3. Anthocyanins

**Figure 4:**
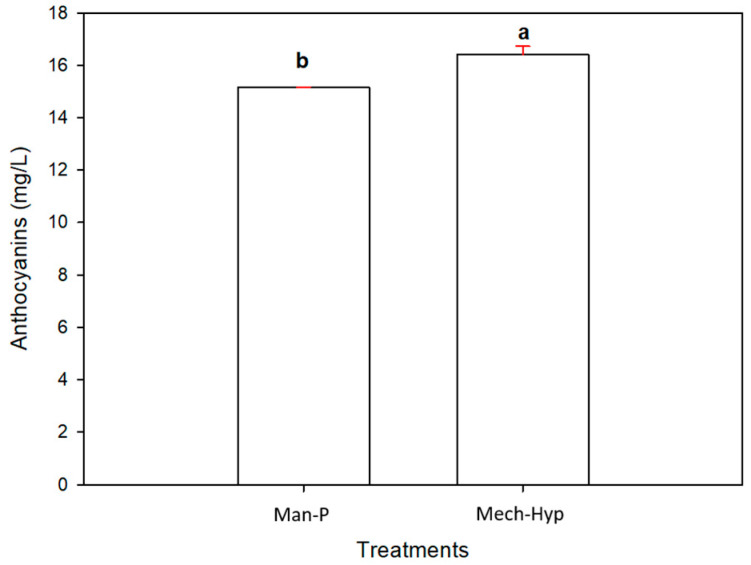
Anthocyanin content in the pomegranate juices. Different letters represent significant differences (*p* ≤ 0.05). Error bars represent ±1 SD of three replicates.

The coloration of pomegranate fruits is due to the presence of bioactive polyphenols such as organic acids, sugars, hydrolysable tannins, punicalicalagin, gallic acid, glucose esters of ellagic acid, flavonoids such as flavones, flavanones, isoflavones, and flavonols, and anthocyanins [5]. The main anthocyanins found mainly in pomegranate fruit are cyanidin 3-mono- and 3,5-diglucosides, delphinidin, and pelargonidin [55]. This study yielded 15.140 mg of cyanidin 3-glucoside/L (mg CyE/L) for the Man-P sample, while Mech-Hyp yielded a higher concentration of 16.409 mg CyE/L, as shown in Figure 4. It was observed that there were significant differences (*p* ≤ 0.05) between the samples due to the method used for juice sanitization. Li and collaborators [54] analyzed juices from pomegranate arils obtained from 10 cultivars from Chinese regions, and reported a concentration of anthocyanins that varied from 0.004 to 0.160 mg CyE/mL. They reported low concentrations of these compounds compared with those obtained in this work. Mena and collaborators [60] analyzed 15 aril juices of Spanish cultivars, the juice was pressed in a nylon mesh with a laboratory pilot press, and the concentration of anthocyanins ranged from 34.2 to 1075 mg CyE/L. In further experimental work reported by Mena and collaborators [43], the juice of the Mollar de Elche and Wonderful varieties from fruits harvested in Alicante had an anthocyanin concentration that ranged between 23 and 136 mg/100 mL, respectively. Kalaycıoğlu and coworkers [61] reported values from 11 to 178 mg CyE/L content in twenty grapefruit cultivars harvested in Yazd Agricultural Research Center, Iran province. Other authors such as Fahmy et al. [55] reported an anthocyanin content of 110–140 mg of cyanidin 3-glucoside equivalents per liter in pomegranate juices. Based on these results, it can be seen that there were significant differences, which may have been due to agronomic factors, temperature, light intensity, genetic factors, processing techniques, and storage conditions of the fruit, all of which influence the anthocyanin content [62]. The use of hyperbaric sanitization compared to pasteurization avoids the degradation of anthocyanins and retains the antioxidant capacity because this technique can be carried out at room temperature without the need to apply heat, unlike pasteurization. Enzymes can be inactivated by applying thermal processing, but the application of heat can cause the loss of bioactive compounds such as anthocyanins [63]. That is why, in this work, the use of hyperbaric sanitization is suggested as another alternative for the sanitization and protection of compounds in pomegranate juices. Regarding the anthocyanin content, the Mech-Hyp treatment presented a higher content of these compounds than the Man-P treatment.

### 3.7. Determination of Scavenging of ABTS and DPPH Radicals

Pomegranate is considered a source of bioactive compounds that stop the oxidation chains of some radicals, such as ABTS and DPPH. The method of action of the ABTS radical occurs through the interaction between the ABTS radical cation and an antioxidative agent. Aguirre-Cruz and co-workers [64] mentioned that antioxidants are classified according to their mechanism of action and are called primary and secondary. The first type is characterized by its breaking of the oxidation chain reaction by donating hydrogen and generating more stable radicals, while secondary antioxidants delay oxidation with the help of other mechanisms such as the elimination of oxygen, regeneration of primary antioxidants, chelation of metals, and repair of hydroperoxides. The elimination of these radicals is identified by a bleaching of absorption spectrum maxima, and they are partially degraded as a result of the reaction with polyphenols [65]. 

**Figure 5:**
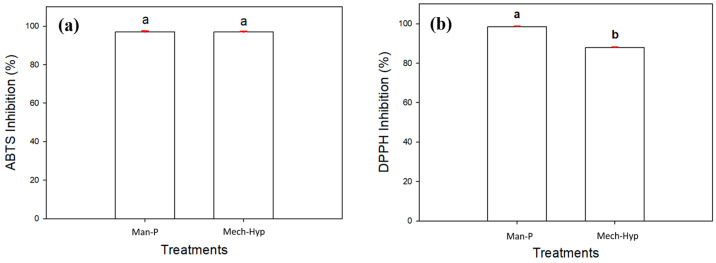
Radical inhibition in juice ABTS (**a**) and DPPH (**b**). Different letters represent significant differences (*p* ≤ 0.05). Error bars represent ±1 SD of three replicates.

Figure 5a shows the results obtained for the antioxidant activity of the samples analyzed in this study. The ABTS radical showed that there were no significant differences (*p* ≥ 0.05) between the treatments, as 96.99% percentage inhibition of the radical occurred in both samples. The results showed a higher percentage inhibition of the ABTS radical than that reported by Bopitiya et al. [37] in aril extracts obtained from various Sri Lankan cultivars; the Nayana cultivar presented the highest value of 93.1%, followed by the Nimali cultivar with 91.2%, Indian with 89.7%, and finally, Daya with 72.7%. The ABTS radical had an initial green color, and the addition of pomegranate juice produced a color degradation due to the presence of polyphenols that captured the radicals and stabilized them. It can be seen that the juices obtained in this work showed greater inhibition than those reported by other authors. 

Regarding the DPPH radical, Figure 5b shows that the highest inhibition occurred in the Man-P sample at 98.48%, followed by the Mech-Hyp sample at 87.93%. The results showed a higher inhibition than that reported by Aloqbi and collaborators [58]. They analyzed the percentage of inhibition of the DPPH radical in pomegranate juice at different concentrations and found values between 14.4% and 37.9%, highlighting that the pomegranate juice with the highest concentration had the highest radical elimination effect. Tezcan and collaborators [40] reported that the inhibition of the DPPH radical was between 10.37% and 67.46% in seven brands of natural juices in a Turkish market. These results were lower than the percentage of inhibition reported in this work. It is important to note that the juices analyzed by Tezcan involved the whole fruit, including the peel. It is well known that there is a greater number of compounds in this part of the fruit such as punicalagin, which is known to possess greater antioxidant activity. In this work, a greater inhibition was observed in the Man-P treatment. This was attributed to the fact that it had a higher phenol content compared to the Mech-Hyp sample.

It was considered that the values of this activity determined with ABTS and DPPH were strongly related to the phenolic content. Esposto and collaborators [39] mentioned that the chemical composition and antioxidant activity of commercial pomegranate juices are directly associated with total phenolic compounds and hydrolysable tannins. Their efficacy depends on several parameters and factors, including the state of the physical system, temperature, structural properties, oxidation-sensitive substrate properties, synergistic effects, and the presence of prooxidant compounds [50]. 

### 3.8. Antihypertensive Analysis 

**Figure 6:**
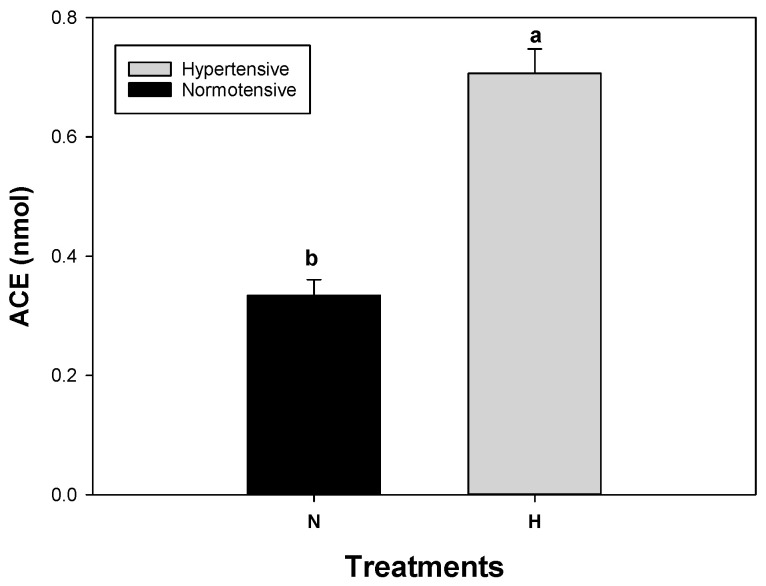
ACE inhibition in hypertensive and normotensive patients. Different letters represent significant differences (*p* ≤ 0.05). Error bars represent ±1 SD of three replicates.

The World Health Organization defines hypertension as a disorder in which the blood vessels have high blood pressure [66]. High blood pressure may be due to sodium homeostasis and fluid retention, and, as a consequence, activation of the renin–angiotensin–aldosterone system [67]. It is estimated that in Mexico, the prevalence of arterial hypertension in vulnerable adults is 49.2%, with 46.8% in women and 52.2% in men. This percentage is considerable, which is why the consumption of healthy foods such as pomegranate juice is chosen to reduce blood pressure in patients with this condition [68]. Figure 6 shows the analysis carried out on the serum used in this study with normotensive (N) and hypertensive (H) people. These data were taken as controls for further analysis. The ACE concentration for the normotensive group was 0.3343 nmol, while the hypertensive group showed an ACE average of around 0.7064 nmol. A previous test was carried out to identify the group of hypertensive people.

**Figure 7:**
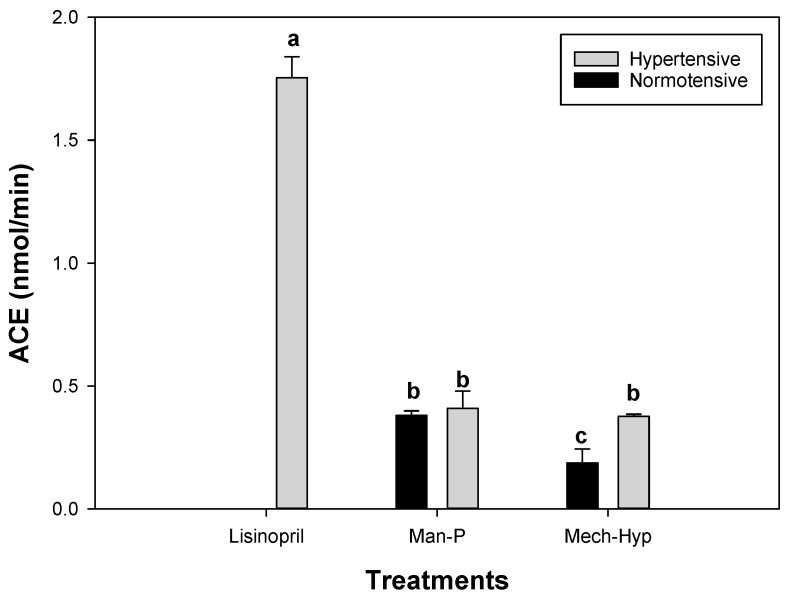
ACE inhibition in pomegranate juice. Different letters represent significant differences (*p* ≤ 0.05). Error bars represent ±1 SD of three replicates.

Figure 7 shows the results obtained for the inhibition of ACE using lisinopril and the treatments evaluated in this research (Man-P and Mech-Hyp). The Man-P treatment showed no significant differences (*p* > 0.05) between the normotensive (0.38 nmol/min) and hypertensive group (0.40 nmol/min). However, the comparison of these results with those of lisinopril showed significant differences (*p* ≤ 0.05). This means that lisinopril inhibited a higher amount of ACE because the drug had a greater inhibitory effect than that of the juice. Looking at the results of the Mech-Hyp treatment, significant differences were observed between the normotensive and hypertensive groups. Serum from the hypertensive group had a better capacity for ACE inhibition (0.37 nmol/min) compared to that from the normotensive group (0.18 nmol/min). This means that pomegranate juice extracted with the mechanical method and sanitized under hyperbaric conditions retained the native properties of the extract. Comparing these data with those obtained in the inhibition of lisinopril, it can be seen that the Mech-Hyp juice was not efficient in the inhibition of ACE. However, comparing the Man-P and Mech-Hyp treatments, the Man-P normotensive serum was found to have significantly higher ACE inhibition (*p* ≤ 0.05) than that of the Mech-Hyp treatment with values of 0.38 nmol/min and 0.18 nmol/min, respectively. Regarding the hypertensive serum, there were no significant differences (*p* > 0.05) between the methods of extraction and sanitization. 

#### Analysis In Vivo

A study by Barati et al. [67] reported that consumption of pomegranate juice for 8 weeks showed beneficial effects on blood pressure, and this effect was attributed to phenolic compounds. In previous studies, 24 healthy volunteers (men and women) consumed one capsule of pomegranate extract for 28 days, and it was observed that systolic and diastolic blood pressure was significantly reduced after consumption [69]. Polyphenols have a hydrophilic and lipophilic domain and can bind to receptors, transcription factors, and enzymes involved in intracellular signaling. These effects allow them to exert biological activity; in addition, antioxidants noticeably reduced hypertension in both human and animal models [70]. According to Aviram and Dornfeld [71], the consumption of pomegranate juice reduces the activity of the angiotensin-converting enzyme and promotes a reduction in systolic blood pressure. This study was carried out among people who ranged from within the range of ages 62 to 77 years old and had blood pressure with average levels. They attributed this effect to the antioxidant capacity of some bioactive components such as tannins. Stockton and co-workers [72] studied the effect of fasting consumption of 100 percent natural encapsulated whole pomegranate extract for 8 weeks on blood pressure in men and women between 18 and 65 years of age. They observed a decrease in diastolic blood pressure and related it to the polyphenols contained in the extract since these reduced angiotensin-converting activity, as well as to the mechanism of polyphenols in increasing nitric oxide levels. Stowe [73] discussed the effects of consuming pomegranate juice on blood pressure and cardiovascular health. He mentioned that pomegranate juice has demonstrated considerable effects as an antioxidant and antihypertensive in humans and mice. In addition, the mechanisms of action of the juice can help to reduce systolic blood pressure, which has a positive effect on the progression of atherosclerosis and coronary heart disease. Treatment for hypertension mainly consists of the use of various drugs, such as captopril, lisinopril, benazepril, and enalapril, among others. These are directed to the renin–angiotensin–aldosterone system, where they exert control over blood pressure by blocking angiotensin receptors or inhibiting ACE [74]. These results suggest that the consumption of pomegranate juice generates an inhibition of ACE due to the benefits of the content of antioxidant compounds. 

## 4. Conclusions

According to this study, it can be concluded that the presence of antioxidant compounds in the juices depends on the sanitization system. It was observed that bromatological analysis demonstrated higher amounts of water, total solids, and carbohydrates in Mech-Hyp treatment due to the mechanical force applied to the extraction of juice. Phenols and antioxidant activity were preserved in greater quantity in the Man-P treatment, while flavonoids and anthocyanins were found in greater quantity in the Mech-Hyp treatment. Additionally, the antihypertensive activity demonstrated that the extraction and sanitization practices maintained the compounds of interest, generating the inhibition of ACE. These results are of great importance since pomegranate juice can provide great health benefits due to its antioxidant and antihypertensive properties.

## Figures and Tables

**Table 1:** Bromatological analysis of the pomegranate juices evaluated.

Samples	Moisture (%)	Total Solids (%)	Fat (%)	Ash (%)	Protein (%)	Carbohydrates (%)
Man-P	${88.81\;\pm \;0.06\;}^{b}$	${11.18\;\pm \;0.06\;}^{a}$	${0.27\;\pm \;0.20\;}^{a}$	${0.20\;\pm \;0.03\;}^{a}$	${0.17\;\pm \;0.01\;}^{a}$	${10.53\;\pm \;0.25}^{\;a}$
Mech-Hyp	${92.83\;\pm \;0.04\;}^{a}$	${13.53\;\pm \;0.08}^{\;b}$	${0.29\;\pm \;0.12}^{\;a}$	${0.26\;\pm \;0.04}^{\;a}$	${0.31\;\pm \;0.19}^{\;b}$	${12.65\;\pm \;0.12\;}^{b}$

Different letters represent significant differences (*p* ≤ 0.05). Values represent ± 1 SD with three replicates.

**Table 2:** CieLab parameters in pomegranate juices.

Samples	L	a*	b*
Man-P	${16.28\pm 0.51\;}^{a}\;$	${15.19\pm 0.41}^{\;a}$	${3.34\pm 0.16\;}^{a}$
Mech-Hyp	${16.91\pm 0.39\;}^{a}$	${13.43\pm 0.37\;}^{b}$	${2.02\pm 0.15\;}^{b}$

Different letters represent significant differences (*p* ≤ 0.05). Values represent ± 1 SD of three replicates.

## Data Availability

The data presented in this study are available on request from the corresponding author. The data are not publicly available because they are part of an ongoing study.
